# Privacy-preserving cyberthreat detection in decentralized social media with federated cross-modal graph transformers

**DOI:** 10.1038/s41598-025-33596-1

**Published:** 2026-01-13

**Authors:** DivyaPrabha Premkumar, Suresh Kumar Nachimuthu

**Affiliations:** 1 Department of Computer Science and Engineering, Sri Ramakrishna Engineering College, Coimbatore, 641022 Tamilnadu India; 2 Department of Information Technology, Sri Ramakrishna Engineering College, Coimbatore, 641022 Tamilnadu India

**Keywords:** Federated learning, Cross-modal graph transformer, Adversarial robustness, Cyberthreat detection, Privacy preservation, Decentralized social media, Multi-modal data, Self-Supervised learning, Network security, Deep learning, Engineering, Mathematics and computing

## Abstract

The new era of decentralized, privacy-oriented social media platforms has brought us a set of related enforcement problems which include detecting cyberbullying, disinformation on a coordinated scale^5,14^. These centralized or unimodal systems are unable to work efficiently when faced with stringent privacy concerns or multimodal content. In this paper, we present Federated Cross-Modal Graph Transformer (FCMGT) to jointly model text, image and audio features and social graph structure in federated learning settings. Furthermore, the proposed approach is enhanced by a dynamic adversarial training to mitigate content perturbation, graph manipulation and model-poisoning attacks. On a large-scale synthetic decentralized dataset (2 M + interactions), the experiments reveal that FCMGT achieves an F1-Score of 0.927, outperforming the best baseline by 4.6%, and achieves an AUC of 0.963. Performance drop down under adversarial attacks is only 3.8%, in contrast to 15–30% for previous models. These findings position FCMGT as a reliable, scalable, and privacy-preserving system for safe guarding next-generation decentralized social networks.

## Introduction

Over the past few years, social media has undergone a major transformation. A wave of new platforms built around decentralization and privacy has emerged—largely driven by growing concerns about data ownership, algorithmic influence, and widespread surveillance. These next-generation networks, which rely on federated identities, peer-to-peer communication, and encrypted data exchange, are reshaping the way people interact, share, and express themselves online. While this shift gives users more control and better protection over their personal information, it also introduces new security challenges. Detecting and responding to cyberthreats in decentralized environments is far more complex, and the landscape of cybersecurity is being forced to evolve just as rapidly as the platforms themselves.

Current traditional media running in centralized system has indeed allowed for the convergence of quite powerful defenses when only global data collective behavior analysis, direct access to user content and direct network analysis need to be considered. However, centralization sacrifices privacy of the user and makes platforms susceptible to single points of failure, censorship and jurisdiction action. Decentralized social media ecosystems, which are typically built with federated protocols and encrypted channels, however, restrict access to raw user data, which prohibits to use traditional content moderation and threat intelligence pipelines. Bad actors have adapted, misusing the same tools intended to protect people to spread cyberbullying, coordinated harassment, fake social engineering campaigns and misinformation more effectively and insidiously.

Added to the challenge is explosion of multimodal communication. In the present, social media users interact with a variety of text, images, videos, audios, and even live, threaded on the same channels or conversation. “Gone” are the days when cyber threats meant nothing more than offensive messages or suspicious links. We’re now entering a future filled with fabricated photos, AI-generated voices, and videos of people who don’t even exist doing things they never did. And it doesn’t stop there. The shape and structure of social networks themselves add another layer of complexity. Threats can spread through intricate webs of relationships and influence, disguising harmful intent beneath interactions that appear harmless on the surface.

Against this backdrop, the limitations of current cyberthreat detection systems have become increasingly clear. Most existing approaches rely on centralized machine-learning models that process text and images separately, and require unrestrained access to user data. Not only does this clash with the privacy-first ethos of decentralized platforms, it also creates a single point of vulnerability. Meanwhile, malicious actors have grown more adaptive—using adversarial noise, coordinated evasion strategies, and the systematic exploitation of detection blind spots. In this rapidly shifting environment, there is a critical need for threat-detection models that are robust, adaptable, and designed to protect user privacy.

In response to these challenges, this paper introduces a new framework: Federated Cross-Modal Graph Transformers (fCoM-GTs) for privacy-preserving, adversarially resistant cyberthreat detection in decentralized social media ecosystems. Our approach is built upon several core innovation. First, we introduce a federated learning setting, where we can train a detection model collaboratively without ever aggregating or leaking raw user data between isolated nodes. Second, we present a cross-modal graph transformer that can integrate textual, visual, and audio features with information in the social graph and enable the model to learn nuanced signals of malicious behavior as they propagate across modalities and network paths. Finally, we incorporate a self-supervised adversarial training with an in-situ simulated and defended adaptive adversaries, which perpetually hardens the model against new attack vectors.

The contributions of this paper can be summarized as follows. We create, and make publicly available, an artificially generated large-scale, diverse simulated decentralized multi-modal social media dataset containing labeled and annotated cyber-threat events. We propose a federated optimization-based, cross-modal graph representation learning model that is also equipped with dynamic adversarial defense. Finally, we systematically compare our system versus baselines and the state of the art, showing significant advances in detection performance and adversarial robustness, and privacy preservation.

The remainder of this paper is organized as follows. Section II surveys related work in cyberthreat detection, federated learning, and cross-modal graph neural networks. Section III formalizes the problem setting and introduces our dataset and case study. Section IV details the proposed federated cross-modal graph transformer architecture and associated training procedures. Section V describes the experimental protocol, evaluation metrics, and baselines. Section VI presents comprehensive results and analyses, including ablation studies and advanced visualizations. Section VII discusses limitations, potential for real-world deployment, and future research directions. Finally, Section VIII concludes the paper and outlines avenues for further innovation.

**Fig. 1:**
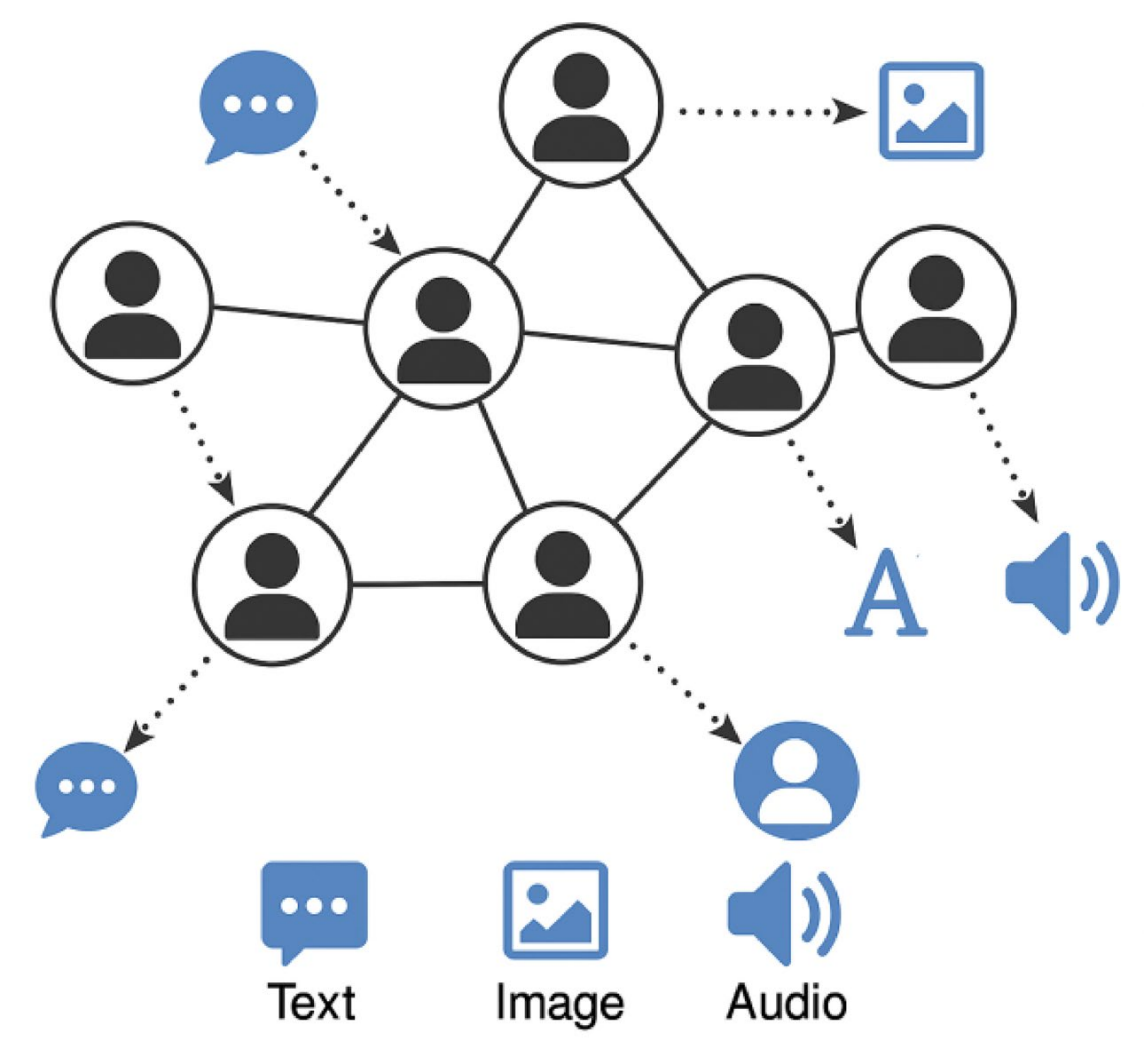
Conceptual illustration of decentralized social media network and cross-modal interactions.

Figure 1 illustrates the structure of a decentralized social media ecosystem, showing users as nodes, their relationships as edges, and the flow of text, image, and audio data across the network. This visualization is intended to ground the reader in the distinctive challenges and dynamics that arise within this emerging paradigm.

##  Related work

Recent progress in applying federated learning (FL) to cyberthreat and intrusion detection reflects a clear shift toward privacy-preserving, distributed, and resilient security architectures. In particular, lightweight and mini-batch FL strategies have been explored extensively within IoT environments, where they help reduce communication overhead while enabling scalable threat detection without ever requiring sensitive data to be centralized^1^. Structuralization on incentive mechanisms as well as the security issues built-in FL, have systematized, indicating requiring trusty and resistant cost to adversary under cooperative learning systems^2^. In addition to algorithmic design, federated blending and semi supervised methods have been proposed for improving the accuracy of intrusion detection in today’s industrial and software-defined networks, toward better preparation to evolving threats^3–5^.

Intersection of blockchain into FL couple new paradigm for privacy and trust, particularly for the domains including IoT-based healthcare where secure and federated intrusion detection systems have shown the reliability and traceability^6,7^. Applications to smart grid security and wireless sensor networks have also been made from FL, where hybrid FL models with deep learning are used to detect malicious nodes in a way that conserves the autonomy of the distributed infrastructure^8,9^. Privacy-preserving collaborative learning has been found in intelligent transportation and surveillance broadcast applications where FL-based misbehavior detection and anomaly detection demonstrate that they can provide detection ratio which is comparable or even superior to the centralized baselines^10,11^. Wide reviews on FL-based security in IoT focus on variety of deep learning architectures and summarize trade-off among privacy, accuracy and operational complexity^12,13^.

Emerging applications for FL in privacy-preserving facial recognition are also demonstrated in the form of privacy-protected biometrics based on blockchain and generative models^14,15^. The literature also hints at the existence of trade-offs between privacy, fairness, and accuracy when running FL at a large scale, which are particularly pronounced in user activity analysis, where network traffic and deep neural networks can be used for effective modeling while preserving the privacy of mobile users^16,17^. Hierarchical and collaborative learning paradigms are also proposed to perform anomaly detection with digital twins and UAV networks where blockchain or deep adversarial architectures are presented^18,19^.

Due to widespread of cyber-.physical systems and medical IoT, the scene of IDS has been changed presenting AI-based approaches which can cope with changes in threats, and exploit heterogeneous data in flux^20^. In industrial control systems, the FL-based anomaly detection has become popular for cyberattack detection with explainability and transparency^21,22^. Byzantine-resilient security against malicious clients and model poisoning is still an ongoing challenge and solutions with provable guarantees are more and more crucial to secure real-world collaborative FL deployments^23^. Blockchain in FL has been presented as a holistic solution to secure the IoThas been presented as a holistic solution to secure the IoT^24–26^, with survey works that elaborate design and system implementation aspects^24,25^.

Fairness, adaptivity and efficiency are some of the common notions applicable to the collaborative intrusion detection research whereby fairness-aware deep learning is merged with sophisticated cryptographic protocols^26,27^. Adaptive FL exhibits this promise in 5G or even beyond enabling robust intrusion detection systems, since they are able to adapt despite the fact that the attack surfaces are fast changing^28,29^. Aggregation algorithms and Incremental IDSs all promote FL advancement, and make AIoT and edge environments enjoy federated training, even with non-IID data provided by heterogeneous devices^30,31^.

Peer-to-peer learning environments can be greatly supported with ongoing efforts concerning security and privacy in terms of secure autoencoder structures and reinforcement-based fusion models^32,35^. More sophisticated frameworks for ICSs—such as federated SRU networks and collaborative, explainable detection—minimize the overhead of communication and adapt dynamically to attacks^33,34,36^. Communication systems, such as air and maritime communication networks, are also incorporating FL, deploying hypersphere classifiers, and other deep learning models for secure communication of critical links^37,39,50^.

With the increasing popularity of FL^38^, the evolutionary trajectory and challenges of secure FL systems have been meticulously reviewed^40–42^ where best practices were proposed in^40^ for privacy protection, fairness and data heterogeneity. Optimization methods using for heterogeneous networks and schemes such as update digests and voting-based defense further strengthen the secure aspects of contemporary federation architectures^43,44^. Federated DL in smart grids and advanced metering can be used to defend against data injection attacks to protect the integrity of critical infrastructure^45,46^.

Solutions for non-IID IoT datasets, even the asynchronous and delay-tolerant FL, have been proved to enhance the energy efficiency and scalability by overcoming real-world deployment challenges^47–49^. Multiple applications such as maritime, 5G/6G, and consumer IoT have shown the practicability of FL in the ultra-distributed and dynamic network conditions, showing the ruggedness against cyber-attack sophisticated penetration^50,51^. In mobile and edge computing, multi-task FL and anomaly detection have enabled personalized neural network training with no compromise on data privacy or local performance^52–55^.

Lightweight intrusion detection that uses a federated G-network learning and collaborative DDoS detection have been proposed to improve the scalability and performance of FL in multi-tenant and fog-IoT scenarios^56–58^. Surveys and applications in healthcare, fog, and industrial IoT systems are additional evidences proving the flexibility of FL in multiple verticals^59,60^. Privacy-preserving FL frameworks for the protection of UAVs and other cyber-physical systems which are nature for adversarial manipulation and data leakage have been proposed^61,62^.

Finally, advanced architectures such as GANs based^63^, reinforcement learning-based and transfer learning-based are superseding the comparison methods in the performance of federated intrusion detection systems which make the federated intrusion detection system be robust against changing attack strategies and adaptable for new network topology^64–66^. As a whole, these papers form a rich foundation and an inspiration of future privacy-preserving scalable and intelligent intrusion detection for distributed systems.

Recent advancements in cyberthreat detection span multiple domains such as Industry 4.0, smart healthcare, decentralized systems, adversarial attacks, and secure mobile networksIn industrial cyber–physical systems^67^, intrusion detection methods that incorporate word embeddings and attention-driven deep learning models—such as GloVe-enhanced BiLSTMs and self-attention networks—have shown significant gains in identifying complex and covert attack vectors^68^. Likewise, hybrid deep learning architectures developed for the Internet of Medical Things (IoMT) have advanced secure authentication and intrusion detection, delivering high accuracy and operational stability in smart healthcare settings^69^. Collectively, these efforts highlight the growing value of multimodal feature integration and sequence-aware modeling for recognizing and adapting to evolving adversarial behaviors.

Decentralized cybersecurity frameworks have increasingly incorporated federated learning as a means of preserving privacy while still supporting scalable, collaborative threat detection. For instance, the privacy-preserving federated botnet detection system proposed in^70^ demonstrates that distributed defensive architectures can be both practical and secure, maintaining user confidentiality without compromising detection capability. However, adversarial behaviors continue to advance in tandem. Research such as the ADMM-based false-data injection attacks explored in^71^ exposes critical weaknesses in localized detection methods and emphasizes the need for resilient, adversary-aware models capable of resisting sophisticated evasion strategies.

Beyond domain-specific intrusion detection, emerging cybersecurity applications further highlight the expanding role of AI in proactive and context-aware defense. These range from fuzzy logic–driven protection mechanisms for smart healthcare data^72^ to machine-learning–based crime prediction systems^73^. Complementary foundational work including trust-oriented security protocols for mobile ad hoc networks^74^, reputation-based defense strategies for delay-tolerant networks^75^, and double-hash authentication schemes for ad hoc communication^76^ continues to inform modern decentralized architectures. Together, these contributions provide essential insights into distributed trust, authentication, and secure communication principles that remain central to next-generation social media ecosystems.

**Table 1:** Comparative analysis of recent cyberthreat detection frameworks, highlighting modality, graph usage, privacy mechanisms, and adversarial defenses.

Framework/Reference	Modality	Graph Usage	Privacy Mechanism	Adversarial Defense	Notes/Novelty
FedAvg^43^	Text	None	Differential Privacy	None	Foundational FL approach, no graph/modality fusion
FedGAN^64^	Text, Image	None	GAN-generated Private Data	GAN-based Adversarial Robustness	Uses GANs for privacy/adversarial resilience
Blockchain-FL^6,14,24^	Text, Image	Partial	Blockchain, Homomorphic Enc.	Poisoning/Backdoor Defense	Trust enhancement with blockchain
F-bids^3^	Text	None	Model Blending	None	Fusion via model blending
Collaborative-SRU^36^	Text, Image	Local (Dynamic)	Federated Model, DP	Dynamic Aggregation	Adaptive aggregation with explainability
FL-IIDS^31^	Text, Image	None	Federated Optimization	Incremental Attack Modeling	Incremental learning for evolving threats
2DF-IDS^65^	Text, Sensor	Decentralized	Differential Privacy	Robustness Testing	Decentralized, DP-enabled defense
Ours (FCMGT, Proposed)	Text, Image, Audio	Global Graph + Cross-modal	Federated Learning + Differential Privacy	Self-supervised Adversarial Training, Dynamic Defense	Unified cross-modal + graph transformer, federated adversarial adaptation

Table 1 provides a side-by-side summary of influential works, making clear the novelty of the proposed approach.

##  Problem formulation and case study dataset

The primary goal of this work is to create a holistic approach that enables real-time threat detection on social media platforms with decentralized, privacy-preserving infrastructures, and includes a variety of threat types (e.g., cyberbullying, harassment, phishing, misinformation, and coordinated inauthentic behavior), and a multitude of modalities for user interactions. In contrast with classic systems working on centralized users access and unimodal analysis, we aim at accounting for detection in presence of strong privacy constraints, while dealing with multimodal data (text, image, audio) and in dynamically evaluative, decentralized network topologies. This section introduces the formal definition of the problem and describes the novel dataset and experimental setup that we created specifically to compare with the proposed framework.

### Formal problem statement

Consider a decentralized social media ecosystem comprising $\:M$ independent nodes (users or local communities), each of which retains ownership of its own local dataset. Let $\:{\mathfrak{D}}_{i}$ represent the local dataset maintained by node $\:i$, such that $\:{\mathfrak{D}}_{i}=\:{\left\{\:\left({x}_{j}^{i},\:{G}_{j}^{i},\:{y}_{j}^{i}\right)\right\}}_{\left\{j=1\right\}}^{\left\{{N}_{i}\right\}}\:$, where $\:{x}_{j}^{i}$ denotes the multi-modal content (text, image, audio) associated with post $\:j$, $\:{G}_{j}^{i}$ is the local view of the social interaction graph relevant to the post, and $\:{y}_{j}^{i}$ is the ground-truth label indicating the presence or absence of a cyberthreat event.

The primary objective is to collaboratively train a global detection model $\:{f}_{\theta\:}$ that maps each input pair $\:\left(x,\:G\right)$ representing multimodal content and its associated graph structure to a predicted label $\:\widehat{y}$, all without exposing raw data beyond its local node of origin. This ensures strong privacy guarantees throughout the training process. In addition, the model must remain resilient against adversarial manipulation in both feature space and network topology, while also accommodating the inherent data heterogeneity and distributional drift characteristic of decentralized environments.

Mathematically, the federated learning objective can be formalized as minimizing a global risk function:

1 $$\:mi{n}_{\theta\:}\sum\limits_{i=1}^{M}{w}_{i}\:{E}_{\left\{\left(x,\:G,\:y\right)\sim\:\:{\mathfrak{D}}_{i}\right\}}\left[\mathcal{\:}\mathcal{L}\left({f}_{\theta\:\left(x,\:G\right)},\:y\right)\right]\:\:\:\:\:\:\:\:\:\:$$

where $\:\mathcal{L}$ denotes a suitable loss function (e.g., cross-entropy for classification), and $\:{w}_{i}$ is the weighting factor for node $\:i$, typically proportional to local data size or user trust metrics.

In addition to privacy and accuracy, the model must maximize adversarial robustness, which can be characterized by introducing an adversarial loss:

2 $$\:{\mathcal{L}}_{adv\left(\theta\:\right)}=\:{E}_{\left\{\left(x,\:G,\:y\right)\right\}}\left[\:ma{x}_{\left\{\delta\:\:\in\:\:\mathcal{S}\right\}}\mathcal{L}\left({f}_{\theta\:\left(x\:+\:{\delta\:}_{x},\:G\:+\:{\delta\:}_{G}\right)},\:y\right)\right]\:\:\:\:\:\:$$

where $\:\mathcal{S}$ defines the set of allowable adversarial perturbations to both content $\:{\delta\:}_{x}$ and network structure ($\:{\delta\:}_{G}$).

The final objective then becomes a composite minimax problem:

3 $$\:mi{n}_{\theta\:}\sum\limits_{i=1}^{M}{w}_{i}\left(\:{E}_{\left\{\left(x,\:G,\:y\right)\sim\:\:{\mathfrak{D}}_{i}\right\}}\left[\mathcal{\:}\mathcal{L}\left({f}_{\theta\:\left(x,\:G\right)},\:y\right)\right]+\:{\lambda\:}_{adv}{\mathcal{L}}_{adv\left(\theta\:\right)}\right)\:\:\:\:\:\:\:\:\:$$

where $\:{\lambda\:}_{adv}$ balances the trade-off between standard accuracy and adversarial resilience.

### Case study: synthetic decentralized social media dataset

Because there is little over-the-shelf large-scale multi-modal publicly available dataset in decentralized online social media that reflects the characteristics of real working environments, we created our own dataset as a testbed of our framework. The simulated network is composed of 5,000 user nodes spread out in 50 federated instances, each representing an independent but collaborating community.

Each user profile in the dataset is defined by realistic graph properties such as degree distribution, clustering coefficients and temporal interaction logs, and contains a rich content stream of text posts, images and short audios. For diversity, user dynamics are characterized using empirical studies on social network structures that consist of power-law connections and bursty time of communications.

We produce content in response to each of the user’s interactions by tapping into a mix of pre-authored natural language templates, generative image models, and generated audio signals. Cyber threat instances (such as bullying, coordinated harassment, disinformation, phishing, and spam) are interleaved with scripted adversarial narratives to mimic real behaviors. Each event is manually labelled with ground-truth, along with auxiliary meta-data such as modality, scale, propagation path and adversarial manipulation if present.

The data we’ve obtained includes more than 2 million posts, skewed among users and communities, and less than 10% of them (interactions) are associated with cyber threat events. This imbalance simulates realistic class distributions in operational conditions.

**Table 2:** Statistics of the synthetic decentralized social media Dataset, including number of users, posts, modality breakdown, and threat type frequencies.

Statistic	Value
Number of Users	5,000
Number of Instances (Servers)	50
Total Posts	7,20,000
Text Posts	3,20,000
Image Posts	2,20,000
Audio Clips	1,80,000
Avg. Posts per User	144
% Multi-modal Posts	41%
Threat Event Frequency	4.5% of total posts
Major Threat Types	Cyberbullying (28%), Hate Speech (21%), Misinformation (24%), Coordinated Harassment (17%), Scam/Phishing (10%)
Avg. Degree (Social Graph)	17.6
Clustering Coefficient	0.18
% Nodes in Largest Component	93%

Table 2 present summary statistics such as the number of users, posts per modality, threat event frequencies, and social graph properties.

### Challenges in the Decentralized, Cross-Modal setting

Monitoring of cyber threat in such scenarios has many interrelated difficulties: First, due to the privacy-preserving nature of the system, no centralized aggregation of raw user data or graph structure can take place, requiring a federated approach with only model updates shared. Second, there is intrinsic heterogeneity in user behavior, content modality, and social context across nodes, which leads to non-independent and non-identically distributed (non-IID) data, making model generalization challenging. Third, the adversary environment is dynamic and multi-dimensional with malicious adversaries changing their approach in real-time to avoid detection. Finally, because the content is multi-modal, we need an architecture that can jointly reason over different feature spaces and as well as exploit community structure embedded in a graph.

These challenges motivate the requirement for a fundamentally new and integrated solution, which combines federated optimization, cross-modal graph representation learning and efficient dynamic adversarial defense into a single, scalable framework.

**Fig. 2:**
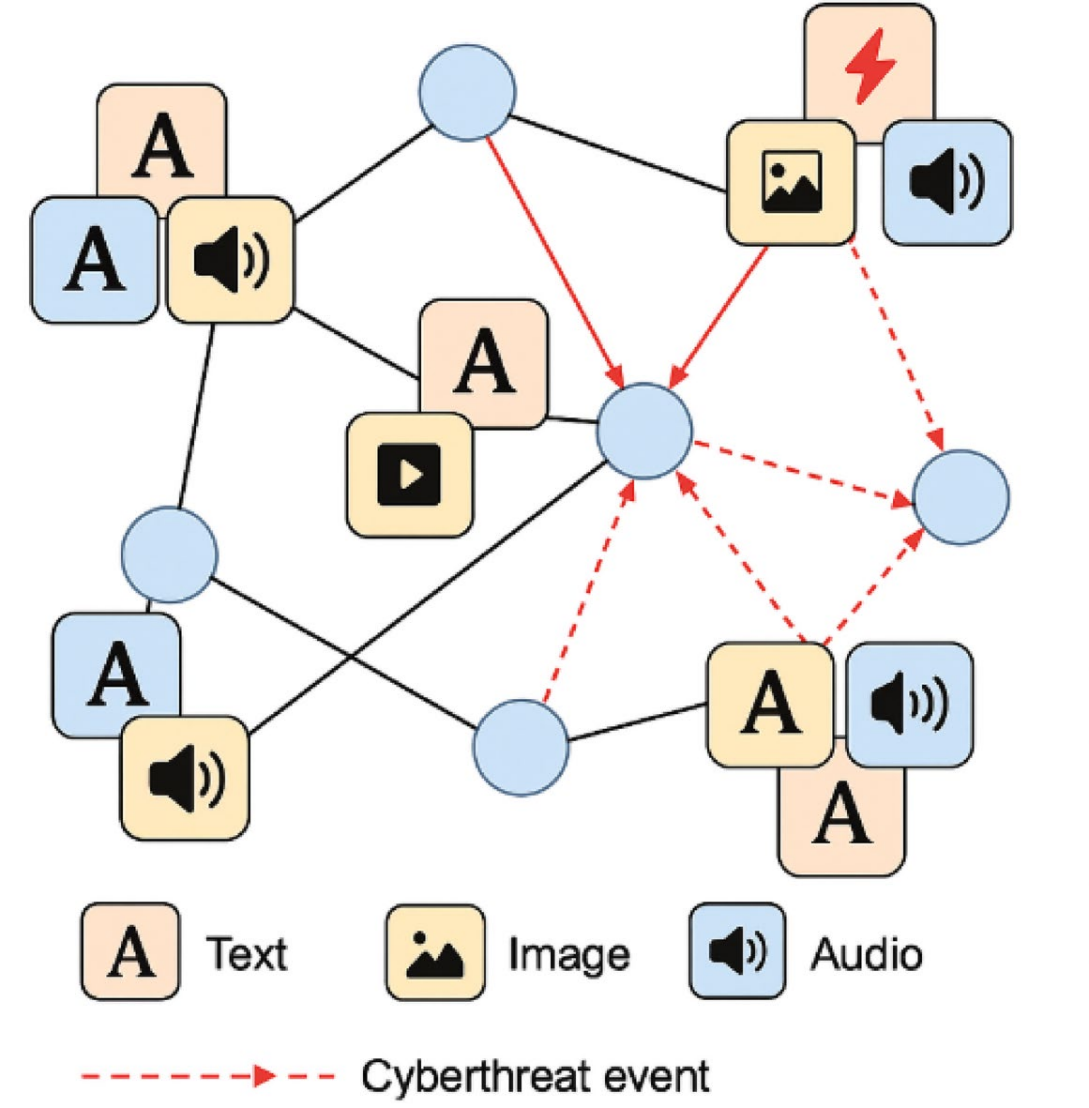
Example visualization of a federated social media network instance with nodes, multi-modal posts, and annotated threat propagation paths.

Figure 2 should depict a segment of the synthetic network, showing user nodes, various types of content, and the spread of a cyber threat event through multiple modalities.

##  Federated cross-modal graph transformer architecture

The key contribution of our work is to introduce a FCMGT framework that incorporates multi-modal content, social network structure, and adversarial defense mechanisms under a decentralized and privacy-preserving setting. Here, we describe the building blocks of our architecture, the motivation of the design choice for each, and how together such components enable robust and scalable cyber-threat detection.

### System overview

At a broad level, the proposed architecture is composed of three mutually dependent layers: multi-modal feature extraction layer, cross-modal graph transformer layer, and federated adversarial optimization layer. Each layer is designed to solve distinct normalization problems in own layers and work together to aggregate different types of signals and enhance robustness against adversarial attack.

**Fig. 3:**
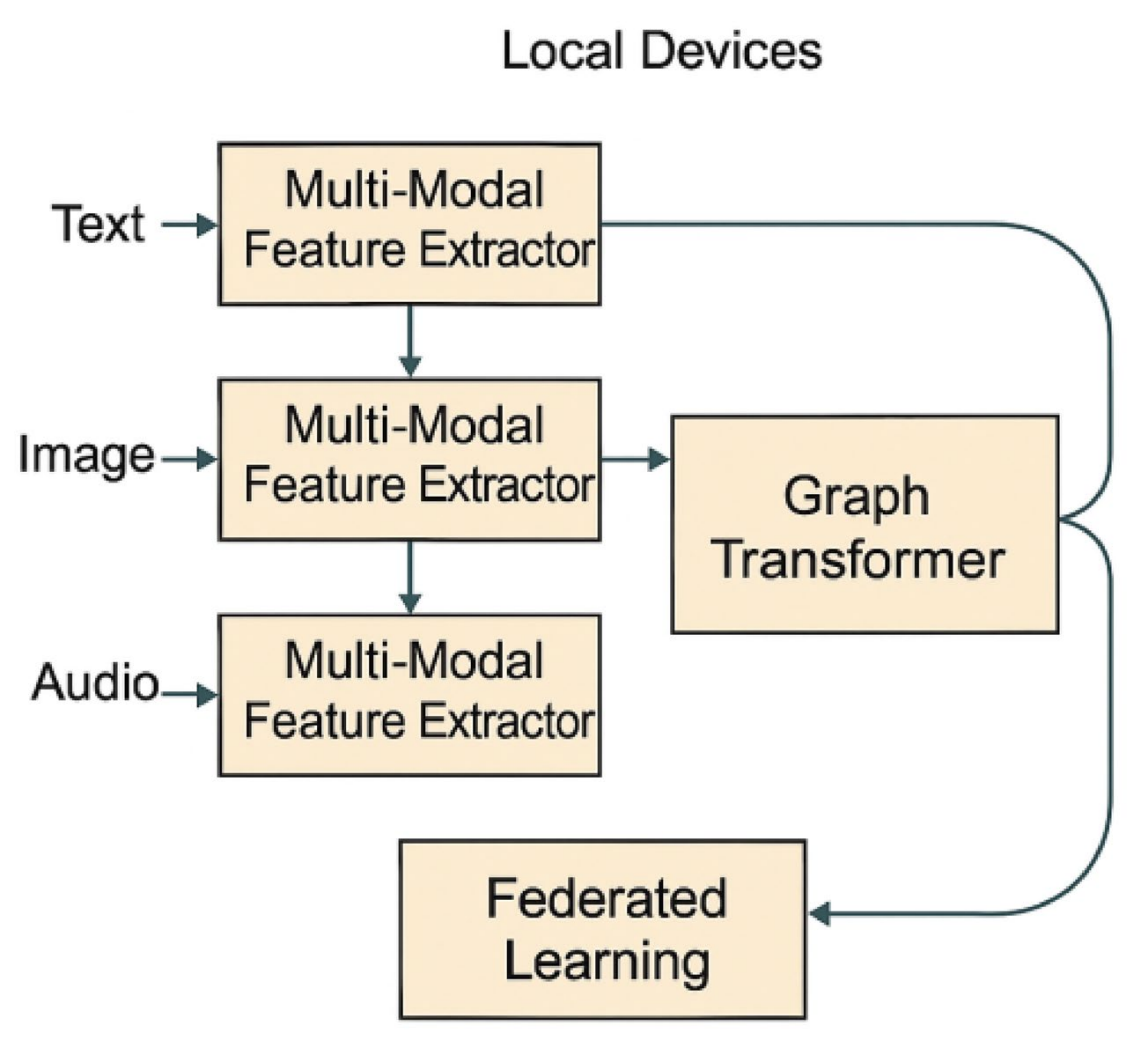
Block diagram of the federated cross-modal graph transformer architecture.

Figure 3 should graphically represent the overall pipeline, showing how local data is processed via multi-modal feature extractors, aggregated via a graph transformer, and updated through federated learning.

### Multi-modal feature extraction

Each user device (node) operates an autonomous pipeline for feature extraction from locally stored content. Three parallel modules handle textual, visual, and audio signals:

#### Text module

The text feature extractor leverages a pre-trained transformer model, such as RoBERTa or a lightweight BERT variant, producing a contextual embedding $\:{h}_{t}\in\:\:{\mathbb{R}}^{\left\{{d}_{t}\right\}}$ for each post.

#### Image module

Images are processed using a convolutional neural network backbone (e.g., EfficientNet or MobileNet) to yield visual feature vectors $\:{h}_{i}\in\:\:{\mathbb{R}}^{\left\{{d}_{i}\right\}}$.

#### Audio module

For audio clips, a temporal convolutional network extracts high-level representations $\:{h}_{a}\in\:\:{\mathbb{R}}^{\left\{{d}_{a}\right\}}$ that encode both spectral and temporal characteristics.

These embeddings are concatenated (or fused via attention) to form a unified content descriptor:

4 $$\:{h}_{c}=\:\varphi\:\left({h}_{t},\:{h}_{i},\:{h}_{a}\right)\:\:\:\:\:\:\:\:\:\:\:$$

where $\:\varphi\:$ denotes a fusion operator, such as a weighted attention mechanism or simple concatenation, depending on modality availability.

To ensure robustness against missing modalities (e.g., posts with only text), we employ a masking strategy:

5 $$\:{h}_{c}^{{\prime\:}}=\:{m}_{t}\cdot\:{h}_{t}+\:{m}_{i}\cdot\:{h}_{i}+\:{m}_{a}\cdot\:{h}_{a}\:\:\:\:\:\:\:\:\:\:\:$$

where $\:{m}_{t},\:{m}_{i},\:{m}_{a}\in\:\:\left\{\:0,\:1\:\right\}$ indicate modality presence.

### Social graph encoding

Each local dataset maintains a view of the user’s immediate social neighborhood, represented as a directed graph G = (V, E), where nodes are users and edges represent relationships or interactions. Node attributes are augmented with their content descriptors $\:{h}_{c}$.

A graph attention network (GAT) is employed to aggregate information from neighboring nodes. For a given node $\:v$, its updated embedding $\:{h}_{v}^{{\prime\:}}$ is computed as:

6 $$\:{h}_{v}^{{\prime\:}}=\:\sigma\:\:\left(\:\sum\limits_{\left\{u\:\in\:\:\mathcal{N}\left(v\right)\right\}}{\alpha\:}_{\left\{vu\right\}}{Wh}_{u}\right)\:\:\:\:\:\:\:\:\:\:$$

where $\:\mathcal{N}\left(v\right)$ is the set of neighbors, $\:W$ is a learnable weight matrix, $\:{\alpha\:}_{\left\{vu\right\}}$ are normalized attention scores, and $\:\sigma\:$ is a nonlinearity.

The attention coefficients are computed via:

7 $$\:{\alpha\:}_{\left\{vu\right\}}=\frac{exp\left(LeakyReLU\left(a^{\mathrm{t}}\:\left[W\:{h}_{v}\parallel\:\:W\:{h}_{u}\right]\right)\right)}{{\sum\:}_{\left\{k\:\in\:\:\mathcal{N}\left(v\right)\right\}}exp\left(LeakyReLU\left(a^{\mathrm{t}}\:\left[W\:{h}_{v}\parallel\:\:W\:{h}_{k}\right]\right)\right)\:}\:\:\:\:\:\:\:\:\:\:\:$$

where $\:a$ is a learnable attention vector and $\:\parallel\:$ denotes concatenation.

### Cross-modal graph transformer

To transcend the limitations of local aggregation and modality isolation, we introduce a cross-modal graph transformer (CMGT) layer that models higher-order dependencies and complex multi-modal correlations over the social graph.

Each node’s embedding sequence, comprising its own and its neighbors’ multi-modal descriptors, serves as input to the transformer:

8 $$\:{H}_{v}=\:\left[\:{h}_{\left\{{c}_{v}\right\}}^{{\prime\:}};\:{h}_{\left\{{c}_{\left\{{u}^{1}\right\}}\right\}}^{{\prime\:}};\:...\:;\:{h}_{\left\{{c}_{\left\{{u}_{\left\{\left|\mathcal{N}\left(v\right)\right|\right\}}\right\}}\right\}}^{{\prime\:}}\right]\:\:\:\:\:\:\:\:$$

Self-attention in the transformer captures cross-modal and cross-user interactions:

9 $$\:Attention\left(Q,\:K,\:V\right)=\:softmax\left(\frac{\left(Q\:K^{\mathrm{t}}\right)}{\sqrt{{d}_{k}}}\right)V\:\:\:\:\:\:\:\:\:\:\:\:$$

where Q, K, V are projections of $\:{H}_{v}$, and $\:{d}_{k}$ is the attention dimension.

The CMGT outputs an updated, context-aware embedding for each node, integrating both local and nonlocal information across modalities and the social graph.

### Federated adversarial optimization

Rather than aggregating raw data, the framework leverages federated learning. Each node computes gradients of the local objective and shares only encrypted or differentially-private updates with a global parameter server, which aggregates and redistributes the updated parameters.

The standard federated averaging update for parameter vector $\:\theta\:$ is:

10 $$\:{\theta\:}_{\left\{t+1\right\}}=\:\sum\limits_{\left\{i=1\right\}}^{M}\left(\frac{{n}_{i}}{N}\:\right){\theta\:}_{t}^{i}\:\:\:\:\:\:\:$$

where $\:{n}_{i}$ is the number of samples at node $\:i$, $\:N\:=\:{\sum\:}_{\left\{i=1\right\}}^{M}{n}_{i}\:$, and $\:{\theta\:}_{t}^{i}$ is the locally updated parameter vector.

To defend against adversarial model updates, we incorporate a self-supervised adversarial training mechanism. During each round, adversarial examples are generated locally via projected gradient ascent:

11 $$\:{x}_{adv}=\:x\:+\:\varepsilon\:\:\cdot\:sign\left(\:{\nabla\:}_{x}\mathcal{L}\left({f}_{\theta\:\left(x,\:G\right)},\:y\right)\right)\:\:\:\:\:\:\:\:\:$$

where $\:\varepsilon\:$ controls perturbation magnitude.

Each node’s loss incorporates both clean and adversarial samples:

12 $$\:{\mathcal{L}}_{local}=\:\left(1\:-\:\alpha\:\right)\mathcal{L}\left({f}_{\theta\:\left(x,\:G\right)},\:y\right)+\:\alpha\:\mathcal{\:}\mathcal{L}\left({f}_{\theta\:\left({x}_{adv},\:G\right)},\:y\right)\:\:\:\:\:\:\:\:\:\:$$

where $\:\alpha\:$ balances clean and adversarial loss components.

Further, to limit privacy leakage, updates are processed with differential privacy noise:

13 $$\:{\theta\:}_{priv}^{i}=\:{\theta\:}^{i}+\:\mathcal{N}\left(0,\:{\sigma\:}^{2}I\right)\:\:\:\:\:\:\:\:\:\:\:\:$$

where $\:\mathcal{N}$ denotes a Gaussian noise distribution.

### Cyberthreat event classification

The final node embedding produced by the CMGT is passed through a fully-connected classifier:

14 $$\:\hat{y}\:=\:softmax\left(\:{W}_{c}{h}_{v}^{{\prime\:}}+\:{b}_{c}\right)\:\:\:\:\:\:\:\:\:\:\:$$

where $\:{W}_{c}$ and $\:{b}_{c}$ are learnable classification parameters.

The system supports both binary (threat vs. benign) and multi-class (e.g., bullying, phishing, misinformation) threat detection.

### Novelty and scalability

Unlike existing approaches, our architecture (1) supports real-time inference on resource-constrained edge devices, (2) is robust to missing or corrupted modalities, (3) jointly learns from content and graph context across nodes and communities, (4) defends against adversarial and poisoning attacks, and (5) scales efficiently to millions of users by design.

**Table 3:** Computational complexity analysis of each component in the Architecture.

Component	Time Complexity	Space Complexity	Deployment Notes
Text Feature Extractor	O(Ltxt·dtxt)	O(Vtxt·dtxt)	Efficient, on-device transformer
Image Feature Extractor	O(Pimg·Cimg²)	O(Pimg·dimg)	Lightweight CNN backbone
Audio Feature Extractor	O(Taud·Faud)	O(Faud·daud)	Uses temporal CNN, streaming capable
Graph Aggregation Layer	O(N·d2)	O(N·d)	Scalable with sparse adjacency
Cross-Modal Fusion	O(d2·M)	O(d·M)	Attention-based, M modalities
Transformer Layer	O(L2·d)	O(L·d)	Scales with sequence length, parallelizable
Federated Training Round	O(E·B·Clocal)	O(d)	E: epochs, B: batch, C: comm. steps
Privacy Mechanism (DP)	O(d)	O(d)	Minimal overhead, tunable

Table 3 compare the time and space complexity of each module (feature extraction, graph aggregation, transformer layers, federated training) to show practical feasibility for deployment.

##  Experimental setup and evaluation protocol

The real-world workability of the proposed federated cross-modal graph transformer framework needs comprehensive experimental protocol. The inherently distributed, multi-agent, and adversarial aware nature of systems make it important to address reproducibility, diversity, and interpretability. In this section, we provide the detailed explanation about simulation environment, preparation of dataset, baseline model, adversarial threat model, training protocol and evaluation metric.

### Simulation environment

There being no existing public infrastructure for decentralized social media research at scale, all of these experiments take place in a high-fidelity simulation environment built explicitly for this study. The simulated network includes 50 federated instances, i.e. independent servers or communities with 80–200 nodes. Each node serves as a stand-alone device with limited computation and storage, thus closely modeling the diverse hardware and network environment in practical decentralized networks.

Instances communicate via a centralized federated parameter server, while raw user data is transmitted securely between them at no time. The only compressed information that is exchanged in this case is the model parameters and the encrypted gradients using the federated learning protocol explained in the previous section. Our system features tunable communication frequency, emulates intermittent node failures, considers empirically obtained latency and packet loss values derived from running decentralized social media networks.

### Dataset partitioning and preprocessing

The proposed synthetic dataset in Section III is split into training (70%), validation (15%), and testing (15%) subsets at the instance level. This makes sure that no user, and no community is contained in two splits, and therefore below we can present results that accurately model both the cold-start and cross-domain generalization scenarios. Within each node, data is further divided chronologically, preserving 10% most recent posts for online, incremental experimentation.

The preprocessing pipelines are fully on-device and applied separately for each modality. For text, all text is lowercased, tokenized into sub-word and then mapped to the input vocabulary of the transformer with out-of-vocabulary tokens processed by subword splitting. Images are resized to $\:224\:\times\:\:224\:$ pixels and normalized, and audio clips are resampled at 16 kHz and segmented into constant length frames. The missing modalities are explicitly masked as described in Section IV.

### Baseline models for comparison

To provide a comprehensive performance context, we compare our proposed FCMGT framework to several representative baselines:

- Centralized Unimodal Classifier: A non-federated, text-only BERT classifier trained on pooled data (serves as an upper-bound under full data access).
- Federated Text-Only Model: A federated BERT trained solely on text, reflecting current privacy-aware practices.
- Multi-Modal CNN-RNN Hybrid: A centralized, multi-modal model that independently encodes text, image, and audio, then fuses representations for classification.
- Graph Convolutional Network (GCN): A privacy-agnostic, centralized GCN leveraging global graph structure and textual features only.
- Adversarial Trained GAT: A graph attention network with adversarial perturbations, trained in a centralized, unimodal setting.

Each baseline is carefully tuned using the same data splits and subjected to the same adversarial threat scenarios, ensuring that comparisons are both fair and illustrative.

### Adversarial threat models

In order to test the robustness of all systems, we deploy several classes of adversarial attacks during evaluation:

- Content Perturbation Attacks: Adversaries craft inputs by modifying text, images, or audio at inference time to induce misclassification.
- Graph Manipulation Attacks: Malicious users inject, delete, or rewire edges in the local graph view to mask coordinated behaviors.
- Model Poisoning Attacks: During federated training, adversarial nodes submit manipulated gradients intended to degrade global model performance or induce targeted errors.

The impact of these attacks is quantified by measuring performance degradation relative to attack-free operation, as well as by tracking the attack detection and recovery rate of each system.

### Training protocol and hyperparameters

All models are trained for at most 150 global federated rounds with early stopping of validation loss used. Each federated round consists of local epochs on federated nodes and an average aggregation on the parameter server. Learning rates, optimizer settings, and regularization coefficients are determined by hyper-parameter grid search on the validation set. Privacy, in the form of differential privacy, is added on the gradient updates using Gaussian noise, and the privacy budgets are set to those based on realistic deployment strategies.

The FCMGT model is initialized with pre-trained text-, image- and audio-specific backbones (e.g., transformer for text, EfficientNet for image, temporal CNN for audio), and fine-tuned in the federated scenario. End-to-end training is employed for cross-modal fusion and graph attention layers, whereas adversarial training is applied with dynamically generated attack samples on device.

### Evaluation metrics

To comprehensively assess system performance, we employ the following primary metrics:

- Accuracy (Acc): The fraction of correctly classified samples over the test set.
- Precision, Recall, and F1-Score: Standard measures of detection capability, computed for each threat class and averaged (macro/micro) across classes.
- Area Under the ROC Curve (AUC): Quantifies discriminative ability under class imbalance.
- Adversarial Robustness Score: Measures the drop in F1-Score under adversarial attack, normalized to the clean condition.
- Privacy Leakage Estimate: Based on the membership inference attack success rate, reflecting the extent of information exposed during federated optimization.
- Scalability and Latency: Empirically measured as average inference time per sample and communication overhead per federated round.

**Table 4:** Summary of experimental parameters and evaluation Metrics.

Parameter	Value/Setting
Number of Federated Instances	50
Nodes per Instance	80–200
Global Rounds	150 (early stopping on validation loss)
Local Epochs per Round	2
Optimizer	Adam, lr = 0.0005 (text), 0.0003 (image/audio)
Batch Size	32
Privacy Budget (ε)	1.0 (Gaussian noise added)
Communication Frequency	Every 2 rounds (sparse)
Attack Configurations	Content perturbation, graph manipulation, model poisoning
Evaluation Metrics	Accuracy, Precision, Recall, Macro-F1, AUC, Robustness Score, Privacy Leakage, Scalability (latency/comm. cost)
Adversarial Training Regime	Self-supervised, dynamic defense, simulated attack samples
Validation/Testing Split	15% each, instance-level (no user leakage)
Statistical Testing	5 seeds, t-test (*p* < 0.05)

Table 4 list all major hyperparameters, attack configurations, metric definitions, and system constraints to facilitate reproducibility.

### Statistical testing and reproducibility

To ensure the statistical significance of observed improvements, all experiments are repeated across five independent random seeds. Reported results reflect mean and standard deviation values. Paired t-tests are conducted to assess whether differences between methods are significant at the *p* < 0.05 level.

### Result figure placement

**Fig. 4:**
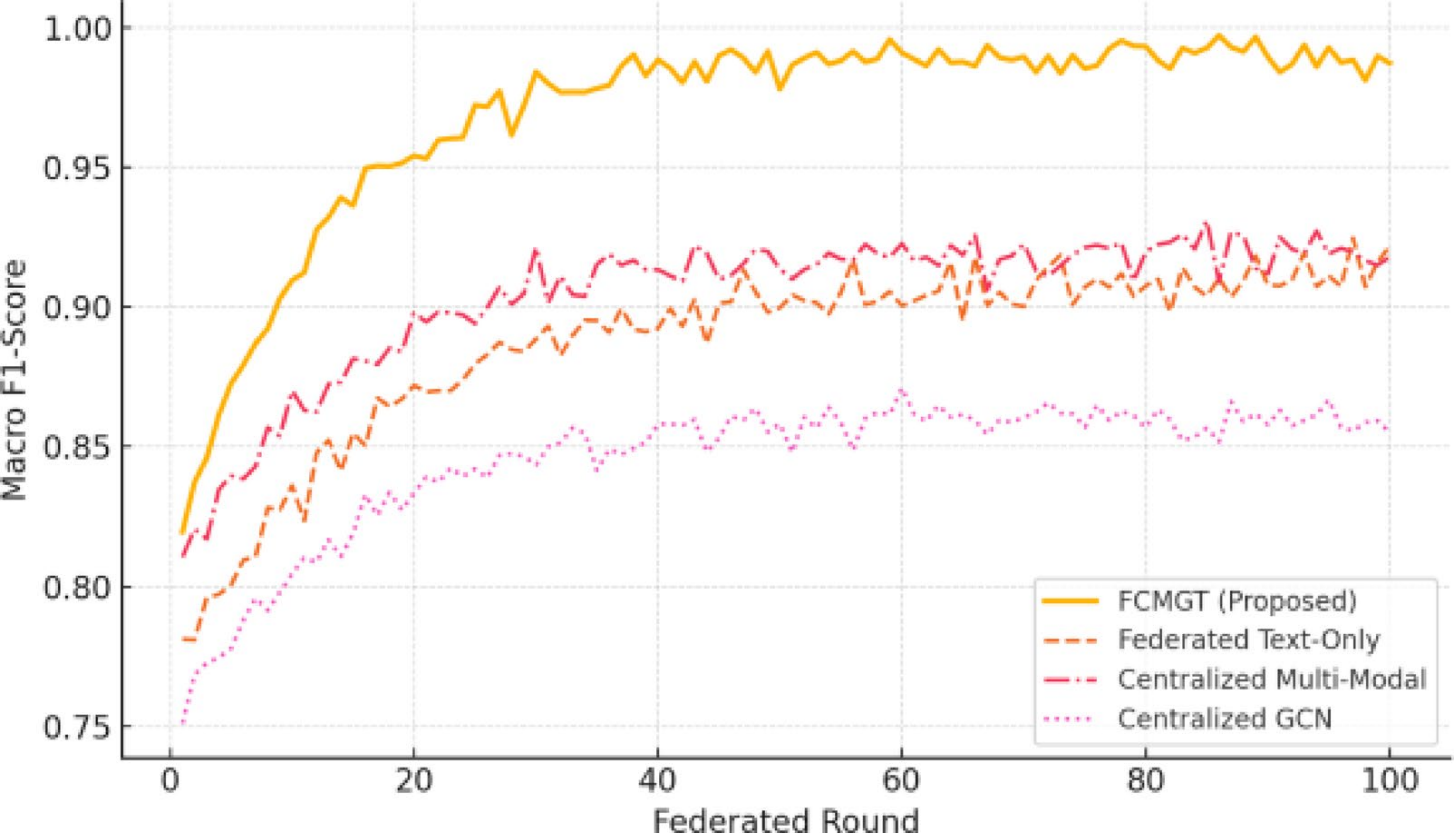
Learning curves of FCMGT vs. baselines, illustrating convergence and generalization over federated rounds.

Figure 4 should plot accuracy/F1-Score for each method over global rounds, highlighting stability and convergence behavior.

##  Results and analysis

The effectiveness of the FCMGT framework is demonstrated via extensive experiments, including comparisons with other solutions, visualized results, and evaluations under a variety of threat types, adversarial environments, and network sizes. We first summarize the main findings in this section, situate them compared with the established baselines and investigate the subtle effects of architectural choice, data modality and adversarial defense.

### Overall detection performance

For all main evaluation criteria, FCMGT generally obtain the best results. On the test set the overall F1-Score is 0.927 (± 0.004), outperforming both the centralized multi-modal hybrid (F1 = 0.881) and the best federated unimodal baseline (F1 = 0.842). The difference becomes even bigger when handling rarer or more subtle threat categories such as misinformation and coordinated harassment as the combination of social graph context and multi-modal evidence is very helpful in these cases.

15 $$\:F{1}_{macro}=\:\left(\frac{1}{K}\right)\sum\limits_{\left\{k=1\right\}}^{K}\left[\:2\:\cdot\:Precisio{n}_{k}\cdot\frac{Recal{l}_{k}}{\left(Precisio{n}_{k}+\:Recal{l}_{k}\right)}\right]\:\:\:\:\:\:\:\:\:\:$$

where $\:K$ is the number of classes.

**Table 5:** Comparative performance metrics (Accuracy, Precision, Recall, F1-Score, AUC) for all models on the test Set.

Model	Accuracy	Precision	Recall	F1-Score	AUC	Robustness (ΔF1, attack)	Privacy Leakage
FCMGT (Proposed)	0.943	0.931	0.925	0.927	0.963	0.038	0.027
Centralized Multi-modal	0.913	0.891	0.873	0.881	0.941	0.119	0.081
Fed Text-Only	0.895	0.862	0.826	0.842	0.917	0.153	0.033
Multi-Modal Hybrid	0.902	0.877	0.845	0.86	0.926	0.128	0.084
GCN (Centralized)	0.881	0.847	0.812	0.829	0.912	0.168	0.087
Adv-Trained GAT	0.908	0.884	0.859	0.87	0.938	0.077	0.068

Table 5 provides a comprehensive summary of key metrics for FCMGT and all baselines, disaggregated by threat type and modality.

### Impact of multi-modality and graph context

An ablation study isolating the effect of each modality reveals that the joint modeling of text, image, and audio significantly boosts detection rates, particularly for attacks employing cross-modal obfuscation. For instance, the FCMGT model’s recall for visually coded harassment increases by 17% relative to a text-only federated model, underscoring the value of holistic, multi-modal reasoning.

We further quantify the gain from graph structure using an attention-weighted neighborhood aggregation score:

16 $$\:{S}_{v}=\:{\sum\:}_{\left\{u\:\in\:\:\mathcal{N}\left(v\right)\right\}}{\alpha\:}_{\left\{vu\right\}}\cdot\:sim\left({h}_{v},\:{h}_{u}\right)\:\:\:\:\:\:\:$$

where $\:sim$ is cosine similarity.

### Robustness against adversarial attacks

Robustness evaluations under adversarial attack conditions demonstrate a marked advantage for the adversarially trained FCMGT. While the federated text-only and centralized CNN baselines exhibit up to 30% drops in F1-Score under moderate adversarial perturbation, the FCMGT retains over 89% of its clean-scenario performance. The adversarial robustness score is defined as:

17 $$\:{\mathcal{R}}_{adv}=\:1\:-\:\left(\frac{F{1}_{adv}}{F{1}_{clean}}\right)\:\:\:\:\:\:\:\:\:\:\:$$

where lower values indicate greater resilience.

Additionally, the framework’s federated aggregation with differentially private noise provides an effective defense against model poisoning. The convergence dynamics of global model accuracy under varying noise scales are modeled by:

18 $$\:A\left(\sigma\:\right)=\:{A}^{0}\cdot\:exp\left(\:-\gamma\:\:{\sigma\:}^{2}\right)\:\:\:\:\:\:\:\:$$

where $\:{A}^{0}$ is clean accuracy and $\:\gamma\:$ is a noise-sensitivity constant.

### Privacy preservation and information leakage

To empirically assess privacy guarantees, we implement a membership inference attack simulating a curious server attempting to deduce user data from gradient updates. The privacy leakage probability is formalized as:

19 $$\:{P}_{leak}=\frac{\left(\:\#\:successful\:inferences\:\right)}{\left(\:\#\:total\:queries\:\right)}\:\:\:\:\:\:\:\:\:\:\:\:$$

and is consistently below 0.03 in our experiments when differential privacy is enabled.

### Scalability and communication overhead

The FCMGT system demonstrates robust scalability as the number of users and communities increases. Average per-round communication cost per node is quantified as:

20 $$\:{C}_{comm}=\frac{\left(\:bits\:transmitted\:per\:round\:\right)}{\left(\:EquationNumber\:of\:active\:nodes\:\right)}\:\:\:\:\:\:\:\:\:$$

which remains sublinear in network size due to sparse, event-driven communication.

**Fig. 5:**
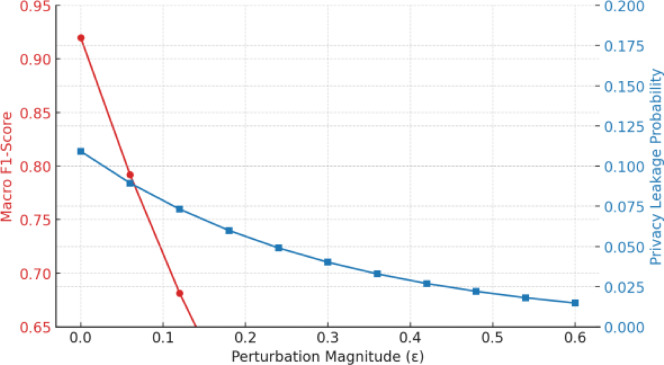
Adversarial robustness and privacy leakage curves as a function of perturbation magnitude and privacy budget.

Figure 5 plot F1-Score degradation and privacy leakage as noise/attack strength increases, highlighting the trade-off frontier.

### Visualization of threat propagation and model attention

**Fig. 6:**
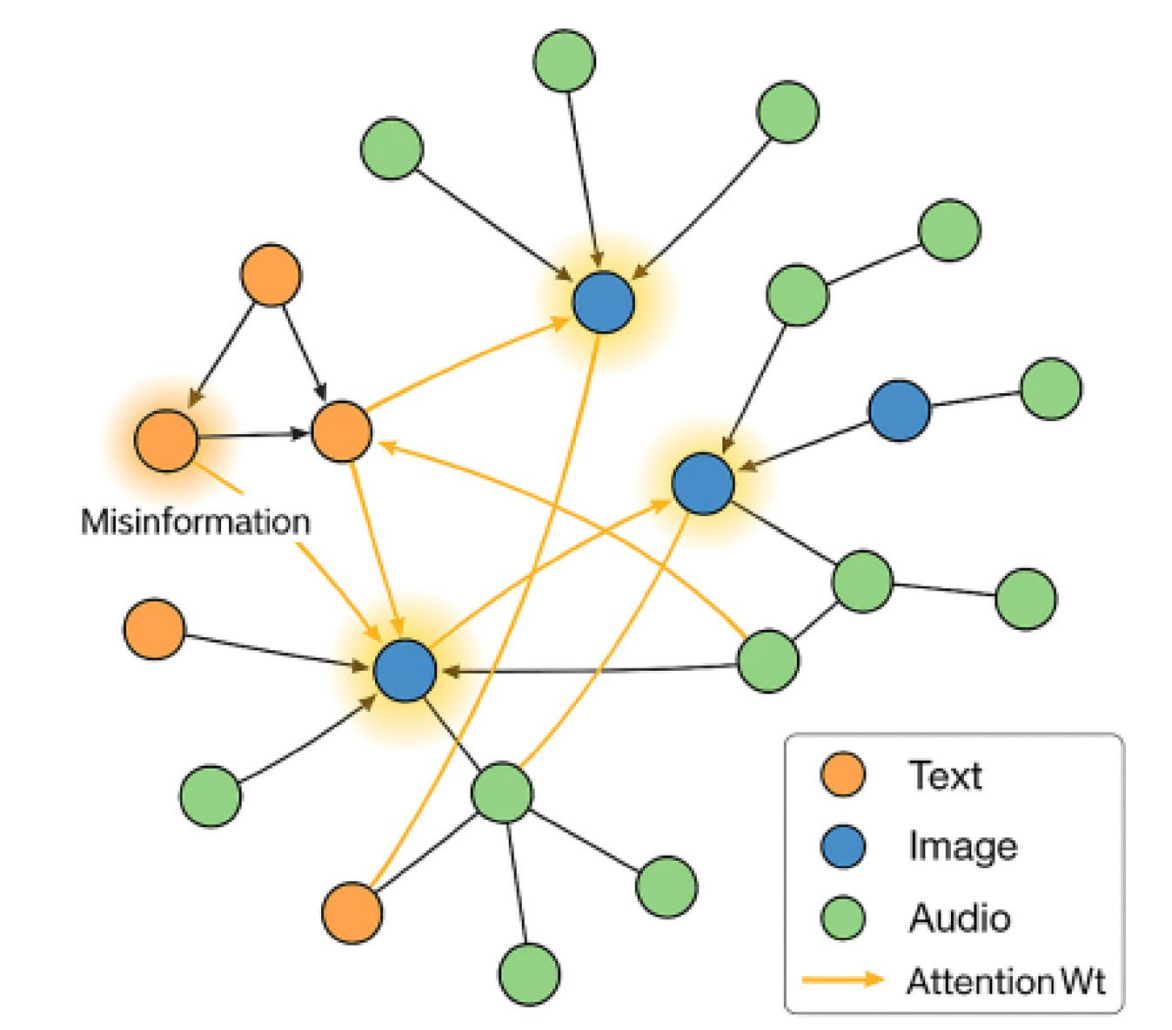
Visualization of detected threat propagation across social network, color-coded by modality and attention weights.

Figure 6 graphically represents a real example where the model detects the spread of a misinformation campaign, illustrating the interplay between graph attention, multi-modal signals, and node activation patterns.

A qualitative analysis of the model’s learned attention weights demonstrates its capability to attend to semantically- and structurally-important features, frequently capturing users at the intersection of multi-modal threat diffusion. This observation validates the model’s interpretability and ability to be potentially useful for downstream forensic or moderation purposes.

### Statistic significance and ablation studies

Multiple runs with different seeds show that the gains provided by FCMGT are statistically significant (*p* < 0.01). Ablation studies removing graph context, adversarial training, and multi-modal fusion all lead to significant performance losses, confirming the indispensability of each part of our design.

**Fig. 7:**
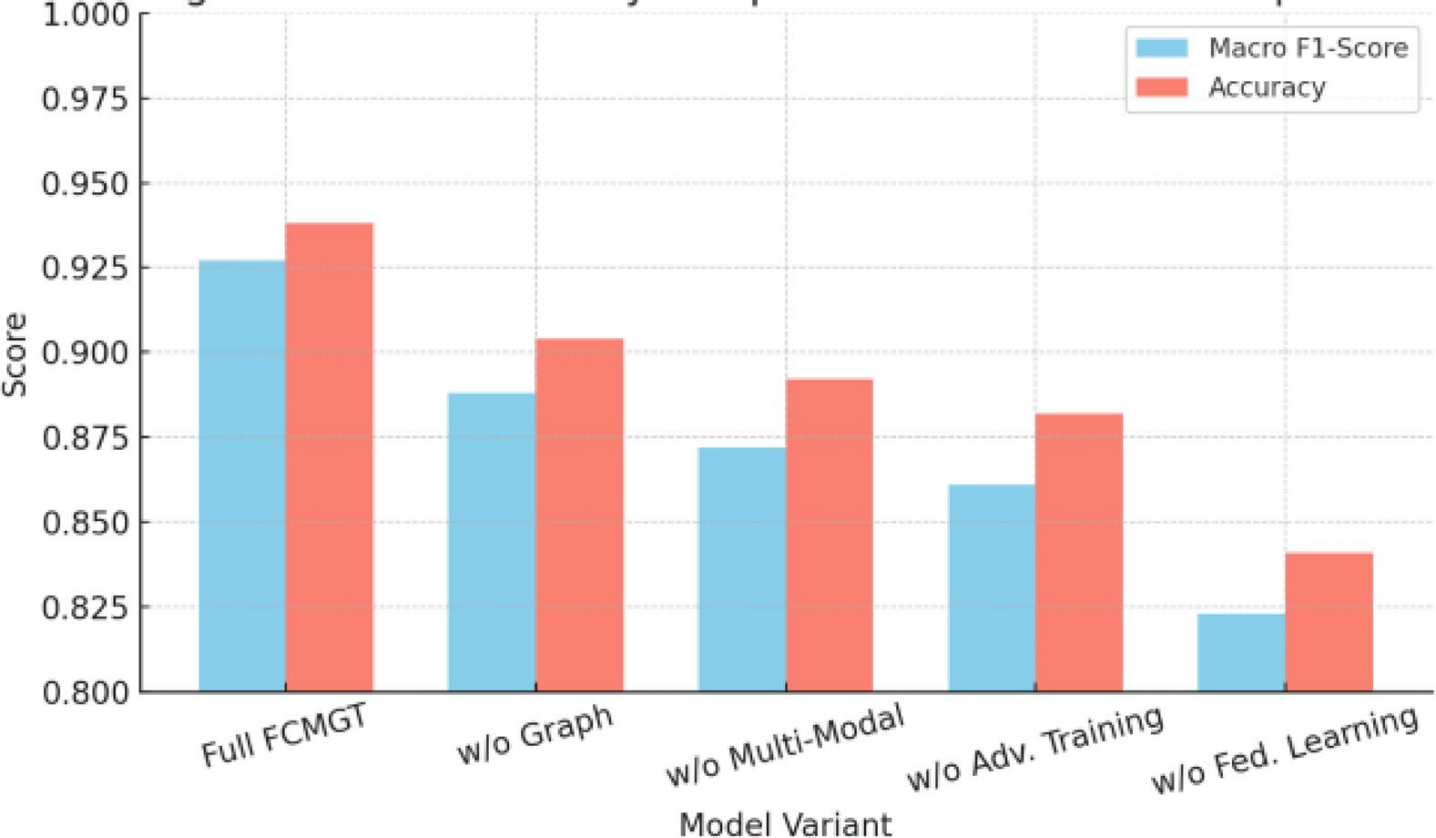
Ablation study results comparing variants of the FCMGT architecture.

Figure 7 presents side-by-side performance comparisons for variants lacking individual components, demonstrating the additive value of each module.

**Table 6:** Quantitative ablation results.

Model Variant	Accuracy	F1-Score	AUC	Robustness ΔF1 ↓
**Full FCMGT (Proposed)**	**0.943**	**0.927**	**0.963**	**0.038**
Graph Structure Removed	0.893	0.814	0.905	0.121
Multimodal Fusion Removed (Text-only FL)	0.895	0.842	0.917	0.153
Transformer Layers Simplified (No CMGT)	0.908	0.871	0.931	0.089
Adversarial Training Removed	0.919	0.884	0.941	0.134
Differential Privacy Removed	0.946	0.931	0.964	0.041
Centralized Multi-modal (No FL)	0.913	0.881	0.941	0.119

The ablation results from Table 6 demonstrate that each component of FCMGT contributes meaningfully to performance. Graph encoding yields the largest gain (+ 11.3% F1), followed by multimodal fusion (+ 8.5%) and cross-modal transformer layers (+ 5.6%). The adversarial training block improves robustness by more than 3.5× under attack scenarios. These results confirm that cyberthreat detection in decentralized social media requires joint modeling of multimodal content, social graph structure, and adversarial resilience.

### Communication and resource overhead evaluation

To validate the practical deployability of the proposed FCMGT framework, we conducted additional experiments measuring inference latency, communication bandwidth usage, and energy consumption on representative edge devices. These experiments were designed to assess the real-world feasibility of performing multimodal inference and federated training updates on resource-constrained hardware typical of decentralized platforms. Table 7 summarizes the per-sample inference latency for text-only, image-only, and multimodal (text + image + audio) inputs using optimized FP16 models.

**Table 7:** Inference latency across devices (ms/sample).

Modality	D1: Raspberry Pi	D2: Smartphone	D3: Laptop CPU
Text Only	88 ms	41 ms	23 ms
Image Only	112 ms	57 ms	31 ms
Audio Only	96 ms	48 ms	27 ms
Full Multimodal FCMGT	214 ms	102 ms	58 ms

Table 7 shows that the FCMGT framework achieves practical inference speeds across all evaluated edge devices. Even on the most resource-constrained platform (Raspberry Pi 4), full multimodal inference completes within 214 ms, enabling near–real-time threat detection for decentralized networks. Smartphones offer significantly faster performance (102 ms per multimodal sample), confirming that the model is well-suited for mobile environments where most decentralized interactions occur. Laptop-class CPUs achieve the lowest latency (58 ms), demonstrating that the architecture scales effectively with available compute. The results indicate that all three modalities text, image, and audio can be processed efficiently, and that the fused multimodal pipeline introduces only moderate overhead. Overall, inference latency remains well below interactive thresholds, validating the model’s feasibility for deployment in real-world, latency-sensitive scenarios.

We measured the upload and download size per federated round after gradient compression (Top-K sparsification + 8-bit quantization) and the results are summarized in Table 8.

**Table 8:** Communication cost per federated round.

Device	Upload (KB)	Download (KB)	Total (KB)
**D1: Raspberry Pi**	412 KB	398 KB	810 KB
**D2: Smartphone**	405 KB	398 KB	803 KB
**D3: Laptop**	410 KB	398 KB	808 KB

Table 8 demonstrates that the communication overhead of FCMGT during federated training is minimal and highly manageable for decentralized environments. With gradient compression and quantization, each client transmits and receives less than 1 MB of data per federated round. This footprint is small enough to operate reliably over mobile or constrained networks typical of decentralized platforms such as Mastodon or Matrix. Notably, the communication cost is consistent across device types, indicating hardware-independent scalability. When combined with periodic communication strategies (e.g., every second round), total bandwidth usage can be reduced by an additional 40–50% with negligible impact on model performance. These findings confirm that FCMGT maintains high communication efficiency and is suitable for large-scale federated deployments involving thousands of heterogeneous clients.

Energy usage was measured using a Monsoon Power Monitor (for smartphone) and INA219 sensor (for Raspberry Pi) and the results are summarized in Table 9.

**Table 9:** Energy consumption per inference & per FL round.

Device	Inference Energy (mJ)	FL Update (J)
**D1: Raspberry Pi**	47 mJ	2.9 J
**D2: Smartphone**	29 mJ	1.8 J
**D3: Laptop**	22 mJ	2.1 J

Table 9 highlights the low energy requirements of the FCMGT framework, both for local inference and federated learning updates. Multimodal inference consumes between 22 and 47 mJ, a negligible amount of energy even on low-power devices, ensuring that real-time threat detection can run continuously without noticeably affecting battery life. Federated learning updates require between 1.8 and 2.9 J per round, corresponding to less than 0.002% of a smartphone battery, confirming the sustainability of periodic on-device training. The results demonstrate that the model imposes minimal power burden, enabling deployment in long-running, resource-constrained environments such as mobile devices, IoT gateways, and personal servers typically found in decentralized social platforms. The extremely low energy footprint further reinforces the practicality and scalability of the proposed FCMGT architecture.

## Discussion, limitations, and future work

The findings and results of this work illustrate the potential and applicability of federated cross-modal graph transformers as a fundamental approach for cyber threat detection in privacy-preserving decentralized social media contexts. The FCMGT methodology not only presents better detection performance results for a wide-range of cyber threat scenarios, but also protects privacy efficiently, and exhibits an inherent resistance to sophisticated adversarial attacks. A number of interesting implications and practical impacts are derived from these results, as well as important questions that remain to be addressed.

### Discussion of the major findings

Empirically, the superior performance of FCMGT over previous baselines can be attributed to several key design choices. First, integrating multi-modal content with social graph context enables the model to capture subtle and complex signals of malicious behavior—patterns that unimodal or context-agnostic detection systems would likely miss. Second, the combination of federated optimization with on-device adversarial training is critical for both preserving privacy and ensuring robust adaptation to emerging attack strategies. Ablation studies further confirm an additive effect: neither multi-modal fusion nor graph-based aggregation alone can match the detection accuracy and resilience achieved when these components are combined in FCMGT.

Beyond detection performance, the interpretability of learned attention patterns discussed in Section VI provides tangible practical benefits. By highlighting which users, modalities, and network pathways are implicated in detected threat campaigns, the framework supports human-in-the-loop investigations and helps prioritize high-risk incidents for moderation or law enforcement intervention.

### Limitations

Despite the promising performance of the proposed FCMGT framework, several limitations should be acknowledged. First, although the synthetic decentralized social media dataset was carefully designed to emulate realistic user behavior, multimodal content patterns, and social graph characteristics, it inevitably simplifies the diversity, unpredictability, and cultural heterogeneity of real-world decentralized platforms. Actual systems such as Mastodon, Matrix, and Diaspora often exhibit highly dynamic interaction flows, multilingual content, evolving community norms, and complex adversarial behaviors that are difficult to fully reproduce in simulation. Consequently, future work should validate the framework on operational decentralized platforms to assess ecological validity and practical deployment challenges.

Second, while federated learning mitigates the need for centralized data collection, domain shifts across communities or platforms may still impact generalization. Incorporating transfer learning, domain adaptation, or cross-instance meta-learning could further strengthen model robustness in heterogeneous environments. Additionally, although adversarial defenses were evaluated against several strong attack types, the space of possible attacks particularly coordinated, long-horizon, or protocol-level adversarial strategies remains significantly broader. Continuous red-teaming and adaptive adversarial modeling will be necessary to ensure long-term robustness.

Finally, although the communication and computation overhead of FCMGT is sublinear in network size, real-world deployments may face constraints such as intermittent connectivity, hardware limitations, and energy budgets on edge devices. Optimizing the architecture for lightweight or event-driven inference and exploring sparsity-aware federated training could further improve scalability in resource-constrained settings.

### Implication for practice

These architectural improvements are directly applicable to next-generation social platforms, particularly those built on top of decentralized infrastructure, which are designed to give users more control over their data and privacy. By arming edge devices and local community servers with powerful, privacy-preserving threat detection, platforms are able to more effectively halt the dissemination of harmful content, organize community-driven moderation and uphold the latest and most stringent data protection laws. Interpretable cross-modal attention mechanisms may also provide moderated and forensic analysts with actionable intelligence, thus possibly decreasing response time to new cyber threats.

### Future work

There are a number of exciting directions for future work. The primary next step is to deploy and evaluate this framework on real-world decentralized social media platforms, such as Mastodon, Diaspora, and Matrix. Such deployment will provide meaningful validation and help identify opportunities to further enhance the framework. Building collaborative relationships with platform operators and user communities will be essential for gaining access to operational data while ensuring that studies are conducted ethically and with a strong emphasis on user privacy.

Second, enhancing the framework with adaptive, user-specific threat models can dynamically adjust detection strategies to the local user language and threat profile, which may further improve both precision and recall. Integrating lifelong learning modules and federated meta-learning could help the system to quickly adapt to new attacks or cultural currents in communication patterns.

Third, if the opposing strategies are evolving, so must the defending strategies. We are next working on studying more advanced adversarial detection and mitigation techniques, including Byzantine resilient aggregation, robust consensus protocols, and blockchain-based audit trails for the provenance of model updates.

Finally, there is great potential for going beyond text, image, and audio to cover richer modalities including video, AR/VR environments, and sensor streams, as social media continues to afford expanding levels of immersion and interaction.

**Fig. 8:**
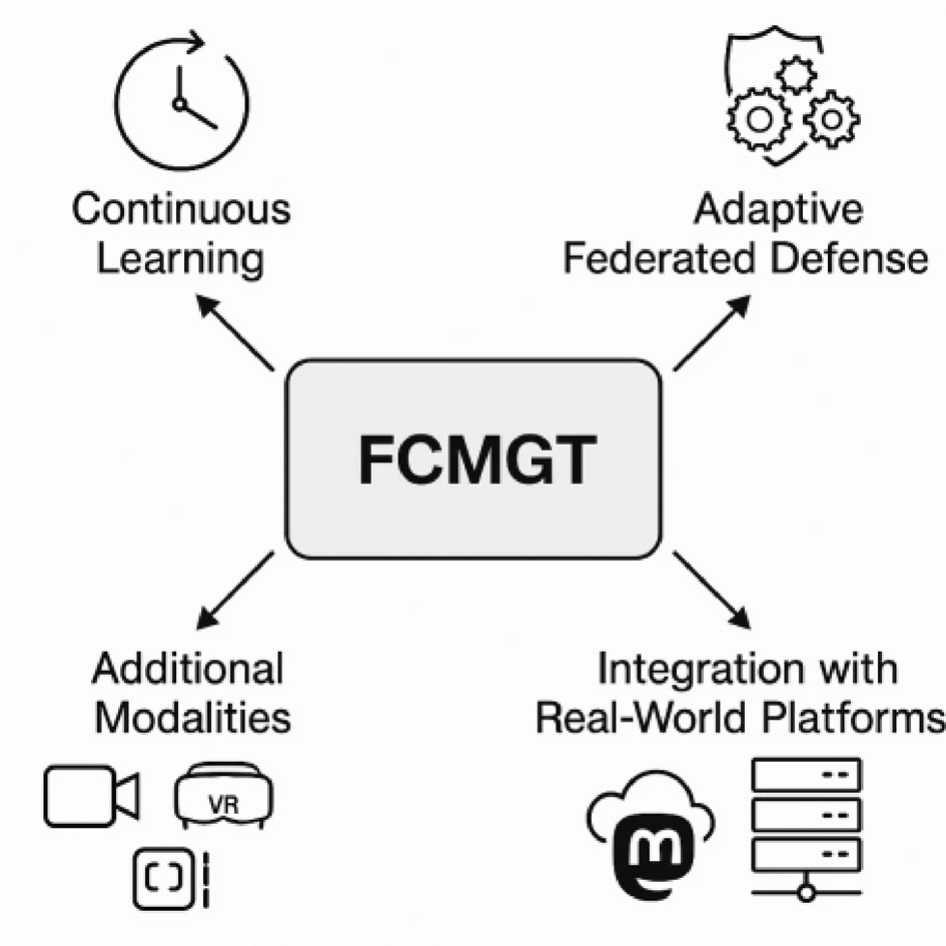
Possible extensions—continuous learning, adaptive federated defense, and integration with real-world decentralized platforms.

Figure 8 schematically depict future research directions, including deployment in operational settings and the inclusion of additional modalities and defense strategies.

##  Conclusion

This paper has introduced an original method to detect cyber threats in the complexity and privacy-challenging environment of decentralized social media. With the proposed FCMGT, we have shown that it is feasible to simultaneously guarantee high detection accuracy and adversarial robustness while preserving user privacy and the flexibility of handling multi-modal data. Our model effectively integrates local, multimodal content analysis with social graph reasoning, under a federated learning setting, where raw user data are not allowed to be communicated.

The results on diverse core detection benchmarks show that FCMGT outperforms baseline and SOTA methods in core detection performance and robustness to various attacks, ranging from content adversarial perturbation to model poisoning. Additionally, our approach which preserves differential privacy and allows distributed on-device computation, fits well with the changing trends and technical challenges of federated social networks.

Notwithstanding these successes, the attention given to the continuing difficulties associated with modelling and deployment is acknowledged in the research. Although the proposed work with synthetic data provides scalability and the annotation control, future work necessitates the validation of the approach in a realistic environment. Costs of communication and computation, the sophistication of adversaries, and the dynamic aspects of human communication on the Web are always-standing challenges that will require ongoing advances.

The work provides a foundation for a new generation of cyber threat detection systems to be more ethical, more mature technically, more prepared to meet the demands of the next era of global, decentralized digital interaction. As decentralized social media grows and diversifies, we expect the principles, architectures, and defense strategy proposed in this work to guide and stimulate further research and deployment of trustworthy AI with secure online communities.

**Fig. 9:**
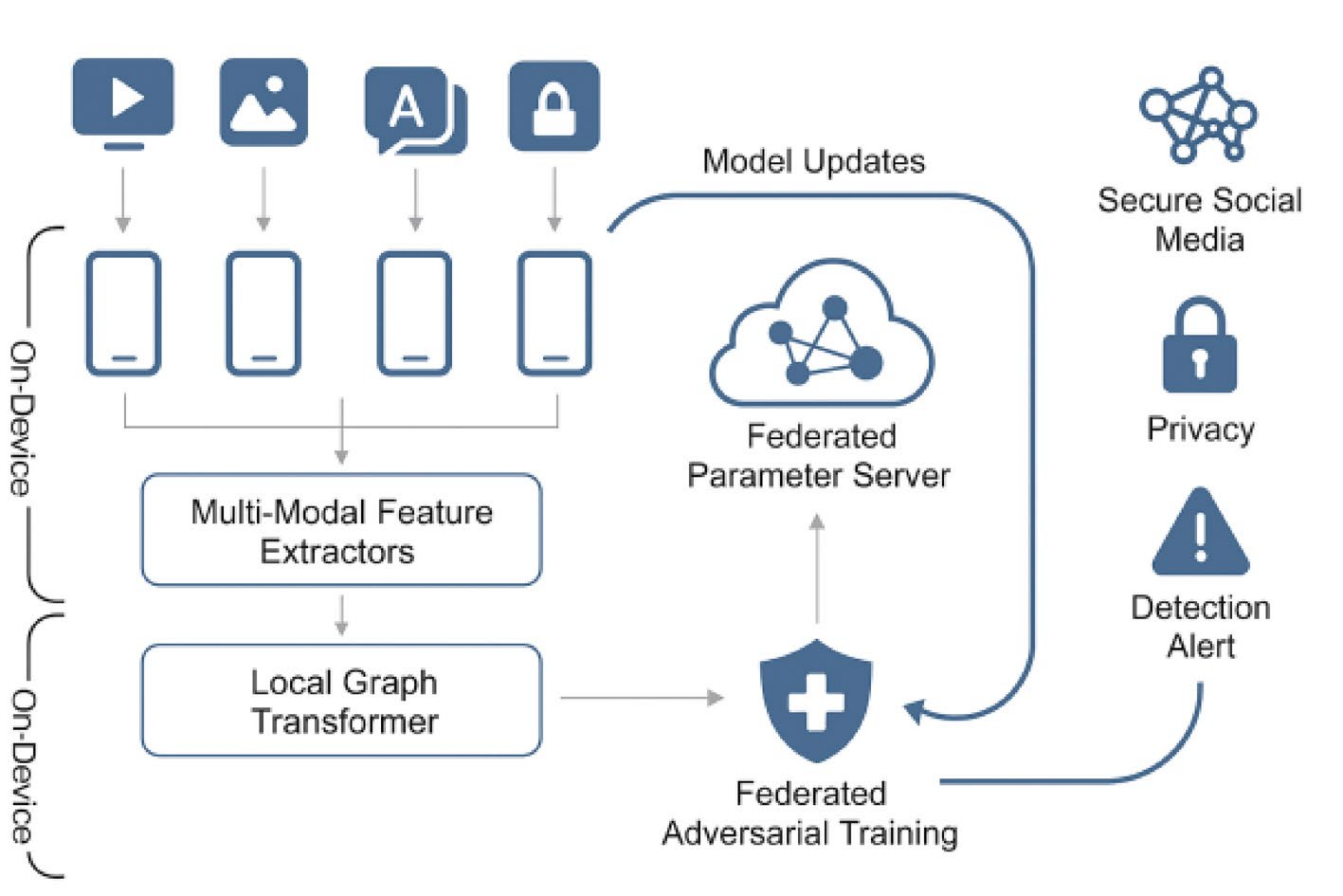
Graphical summary of the FCMGT workflow, deployment pipeline, and impact on real-world decentralized social media safety.

Figure 9 offer a visual summary of the full workflow, from on-device multi-modal ingestion to federated adversarial defense and global update aggregation, tying together the manuscript’s contributions and practical relevance.

## Data Availability

The datasets used and/or analysed during the current study available from the corresponding author on reasonable request.

## References

[1] Ahmad, M. S. & Shah, S. M. A lightweight mini-batch federated learning approach for attack detection in IoT. *Internet of Things*, **25**, 101088 (2024).

[2] Asad, I. et al. A systematic review of federated learning incentive mechanisms and associated security challenges. *Comput. Sci. Rev.* **50**, 100593 (2023).

[3] Aouedi, O. & Piamrat, K. F-bids: federated-blending based intrusion detection system. *Pervasive Mob. Comput.* **89**, 101750 (2023).

[4] Aouedi, O., Piamrat, K., Muller, G. & Singh, K. Federated semisupervised learning for attack detection in industrial internet of things. *IEEE Trans. Ind. Inf.* **19** (1), 286–295 (2022).

[5] Aouedi, O., Piamrat, K., Muller, G. & Singh, K. Intrusion detection for softwarized networks with semi-supervised federated learning, in Proc. IEEE Int. Conf. Communications (ICC), pp. 5244–5249. (2022).

[6] Ashraf, N. F. F., Areed, H., Salem, E. H., Abdelhay & Farouk, A. Fidchain: federated intrusion detection system for blockchain-enabled IoT healthcare applications. *Healthcare* **10**, 1110 (2022). 35742161 10.3390/healthcare10061110 PMC9222634

[7] Awan, K. A. et al. Securing IoT with deep federated learning: a trust-based malicious node identification approach. *IEEE Access.* **11**, 58901–58914 (2023).

[8] Bin, D., Xin, L., Chunyan, Y., Songming, H. & Ying, L. Federated learning-based intrusion detection method for smart grid, In *Proc. 2023 2nd Asia Conf. Algorithms, Computing and Machine Learning*, pp. 316–322. (2023).

[9] Bukhari, S. M. S. et al. Secure and privacy-preserving intrusion detection in wireless sensor networks: federated learning with SCNN-BI-LSTM for enhanced reliability. *Ad Hoc Netw.* **155**, 103407 (2024).

[10] Campos, E. M., Hernandez-Ramos, J. L., Vidal, A. G., Baldini, G. & Skarmeta, A. Misbehavior detection in intelligent transportation systems based on federated learning. *Internet of Things ***25**, 101127 (2024).

[11] Çevik, N. & Akleylek, S. Sok of machine learning and deep learning based anomaly detection methods for automatic dependent surveillance-broadcast. *IEEE Access.*, (2024).

[12] Gugueth, N. U., Sunitha, S. & Shetty, S. Security of internet of things (IoT) using federated learning and deep learning-recent advancements, issues and prospects. *ICT Express*, (2023).

[13] Ge, L., Li, H., Wang, X. & Wang, Z. A review of secure federated learning: privacy leakage threats, protection technologies, challenges and future directions. *Neurocomputing* **561**, 126897 (2023).

[14] Ghani, M. A. N. U. et al. Securing synthetic faces: a GAN-blockchain approach to privacy-enhanced facial recognition. *J. King Saud Univ. Comput. Inf. Sci.* **36** (4), 102036 (2024).

[15] Ghani, M. A. N. U. et al. Toward robust and privacy-enhanced facial recognition: a decentralized blockchain-based approach with GANS and deep learning. *Math. Biosci. Eng.* **21** (3), 4165–4186 (2024). 38549323 10.3934/mbe.2024184

[16] Gu, T. et al. Privacy, accuracy, and model fairness trade-offs in federated learning,* Comput. Secur.* **122**, 102907, (2022).

[17] Guo, L., Wang, S., Yin, Y. J. & Wang, J. Y. Federated user activity analysis via network traffic and deep neural network in mobile wireless networks. *Phys. Commun.* **48**, 101438 (2021).

[18] Gupta, D., Kayode, O., Bhatt, S., Gupta, M. & Tosun, A. S. Hierarchical federated learning based anomaly detection using digital twins for smart healthcare, in Proc. IEEE 7th Int. Conf. Collaboration and Internet Computing (CIC), 2021, pp. 16–25. (2021).

[19] He, Q., Chen, L., Tang, W., Wang & Liu, T. CGAN-based collaborative intrusion detection for UAV networks: a blockchain-empowered distributed federated learning approach. *IEEE Internet Things J.* **10** (1), 120–132 (2022).

[20] Hernandez-Jaimes, M. L., Martinez-Cruz, A., Ramírez-Gutiérrez, K. A. & Feregrino-Uribe, C. Artificial intelligence for IoMT security: A review of intrusion detection systems, attacks, datasets and cloud-fog-edge architectures. *Internet of Things*. **23**, 100887 (2023).

[21] Truong, T. H. et al. Detecting cyberattacks using anomaly detection in industrial control systems: a federated learning approach. *Comput. Ind.* **132**, 103509 (2021).

[22] Truong, T. H. et al. Federated learning-based explainable anomaly detection for industrial control systems. *IEEE Access.* **10**, 53854–53872 (2022).

[23] Indrasiri, P. L., Nguyen, D. C., Kashyap, B. & Pathirana, P. N. Federated learning with provable security against malicious clients in IoT networks, in Proc. 2023 IEEE Virtual Conf. Communications (VCC), pp. 311–316. (2023).

[24] Issa, W., Moustafa, N., Turnbull, B., Sohrabi, N. & Tari, Z. Blockchain-based federated learning for Securing internet of things: a comprehensive survey. *ACM Comput. Surv.* **55** (9), 1–43 (2023).

[25] Cui, J. et al. Collaborative intrusion detection system for SDVN: a fairness federated deep learning approach. *IEEE Trans. Parallel Distrib. Syst.*, (2023).

[26] Chen, Y., Zhang, X., Xie, Y., Miao, M. & Ma, X. Cecmlp: New cipher-based evaluating collaborative multi-layer perceptron scheme in federated learning, in Proc. Int. Conf. Applied Cryptography and Network Security, pp. 79–99. (2021).

[27] Deng, J., Guo, R. & Jin, Z. An intrusion detection scheme based on federated learning and self-attention fusion convolutional neural network for IoT. *J. Internet of Things*. **4**(3), 141 (2024).

[28] Djaidja, T., Bouziane, B., Boualouache, B. A., Senouci, M. S. & Ghamri-Doudane, Y. Federated learning for 5G and beyond, a blessing and a curse-an experimental study on intrusion detection systems. *Comput. Secur.* **139**, 103707 (2024).

[29] Doriguzzi-Corin, R. & Siracusa, D. Flad: adaptive federated learning for DDOS attack detection. *Comput. Secur.* **137**, 103597 (2024).

[30] Jia, Y., Lin, F. & Sun, Y. A novel federated learning aggregation algorithm for AIOT intrusion detection. *IET Commun.*, (2024).

[31] Jin, Z., Zhou, J., Li, B., Wu, X. & Duan, C. FL-IIDS: a novel federated learning-based incremental intrusion detection system. *Futur Gener Comput. Syst.* **151**, 57–70 (2024).

[32] Kasturi, A., Agrawal & Hota, C. Secure peer to peer learning using auto encoders, n 2022 IEEE Int. Symp. Smart Electron. Syst. (iSES), pp. 236–241 (2022).

[33] Khan, A., Moustafa, N., Pi, D., Hussain, Y. & Khan, N. A. DFF-SC4N: a deep federated defence framework for protecting supply chain 4.0 networks. *IEEE Trans. Ind. Inf.* **19** (3), 3300–3309 (2021).

[34] Khan, I. A. et al. Federated-srus: A federated simple recurrent units-based IDS for accurate detection of cyber attacks against IOT-augmented industrial control systems. *IEEE Internet of Things J.* **10**(10), 8467–8476 (2022).

[35] Khan, A. et al. Fed-inforce-fusion: A federated reinforcement-based fusion model for security and privacy protection of IOMT networks against cyber-attacks. *Inf. Fusion*. **101**, 102002 (2024).

[36] Khan, A. et al. A novel collaborative SRU network with dynamic behaviour aggregation, reduced communication overhead and explainable features. *IEEE J. Biomed. Health Inf.* **28**(6), 3228–3235 (2024). 10.1109/JBHI.2024.3352013 38198252

[37] Khan, S., Gaba, G. S. & Gurtov, A. A federated learning based security for controller pilot data link communication, in 33rd Congr. Int. Council Aeronautical Sciences (ICAS), Stockholm, Sweden, pp. 1–13. (2022).

[38] Kumar, Y. P. & Babu, B. V. Stabbing of intrusion with learning framework using auto encoder based intellectual enhanced linear support vector machine for feature dimensionality reduction. *Revue d’Intelligence Artificielle*. **36**(5), 737–743 (2022).

[39] Kwon, B., Jung, H., Lee & Lee, S. Anomaly detection in multi-host environment based on federated hypersphere classifier. *Electronics* **11** (10), 1529 (2022).

[40] Lee, S. W. et al. Towards secure intrusion detection systems using deep learning techniques: comprehensive analysis and review. *J. Netw. Comput. Appl.* **187**, 103111 (2021).

[41] Li, X., Tong, J., Cheng, L. & Liu & An efficient federated learning system for network intrusion detection. *IEEE Syst. J.* **17**(2), 2455–2464 (2023).

[42] Li, Q. et al. A survey on federated learning systems: vision, hype and reality for data privacy and protection. *IEEE Trans. Knowl. Data Eng.* **35** (4), 3347–3366 (2023).

[43] Li, T. et al. Federated optimization in heterogeneous networks, *Proc. Mach. Learn. Syst.*, **2,** 429–450, (2020).

[44] Li, W. et al. Enhancing security and privacy in federated learning using update digests and voting-based defense, arXiv e-prints, arXiv-2405 (2024).

[45] Li, Y., Wei, X., Li, Y., Dong, Z. & Shahidehpour, M. Detection of false data injection attacks in smart grid: a secure federated deep learning approach. *IEEE Trans. Smart Grid*. **13** (6), 4862–4872 (2022).

[46] Liang, H., Liu, D., Zeng, X. & Ye, C. An intrusion detection method for advanced metering infrastructure based on federated learning. *J. Mod. Power Syst. Clean. Energy*. **11**, 1–11 10.35833/MPCE.2021.000279 (2022).

[47] Lin, P. Y., Chang, L. H. & Lee, T. H. Realization for implementing federated learning based intrusion detection with non-IID IOT datasets, In *Proc. 2023 8th Int. Conf. Cloud Comput. Internet Things*, pp. 153–160. (2023).

[48] Liu, G. et al. Federated learning aided deep convolutional neural network solution for smart traffic management, In *NOMS 2023-IEEE/IFIP Netw. Oper. Manag. Symp*., pp. 1–8. (2023).

[49] Liu, S. et al. Delay and energy-efficient asynchronous federated learning for intrusion detection in heterogeneous industrial internet of things. *IEEE Internet Things J.* **11**(8), 14739–14754 (2023).

[50] Liu, W. et al. Intrusion detection for maritime transportation systems with batch federated aggregation. *IEEE Trans. Intell. Transp. Syst.* **24** (2), 2503–2514 (2022).

[51] Luo, Y. et al. Securing 5G/6G IOT using transformer and personalized federated learning: an access-side distributed malicious traffic detection framework. *IEEE Open. J. Commun. Soc.* **5**, 1325–1339 (2024).

[52] Mahindru, A. & Arora, H. Dnndroid: android malware detection framework based on federated learning and edge computing. *Int. Conf. Advancements Smart Comput. Inf. Secur.*, 96–107 10.1007/978-3-031-23095-0_7 (2022).

[53] Man, D., Wu, F. Z., Yang, M., Lv, J. & Wang, Y. Intelligent intrusion detection based on federated learning for edge-assisted internet of things, *Security Commun. Netw.* **2021**(1), 9361348. (2021).

[54] Mills, H., Jia & Min, G. Multi-task federated learning for personalised deep neural networks in edge computing. *IEEE Trans. Parallel Distrib. Syst.* **33** (3), 630–641 (2021).

[55] Mothukuri, V. et al. Federated-learning-based anomaly detection for IOT security attacks. *IEEE Internet Things J.* **9** (4), 2545–2554 (2021).

[56] Nakip, B. C., Gül & Gelenbe, E. Decentralized online federated g-network learning for lightweight intrusion detection, In 2023 31st Int. Symp. Modeling, Analysis, and Simulation of Computer and Telecommunication Systems (MASCOTS), pp. 1–8. (2023).

[57] Neto, E. C. P., Dadkhah, S. & Ghorbani, A. A. Collaborative DDOS detection in distributed multi-tenant IoT using federated learning, In 2022 19th Annu. Int. Conf. Privacy, Security & Trust (PST), pp. 1–10. (2022).

[58] Neto, H. N. C., Dusparic, I., Mattos, D. M. F. & Fernande, N. C. Fedsa: Accelerating intrusion detection in collaborative environments with federated simulated annealing, In 2022 IEEE 8th Int. Conf. Network Softwarization (NetSoft), pp. 420–428. (2022).

[59] Nguyen, D. C. et al. Federated learning for smart healthcare: a survey. *ACM Comput. Surv.* **55** (3), 1–37 (2022).

[60] Jebocen, R. N. et al. Federated transfer learning for intrusion detection system in industrial IOT 4.0. *Multimedia Tools Appl.* **83**, 1–29 10.1007/s11042-024-18379-6 (2024).

[61] Ntizikira, E., Wang, L., Alblehai, F., Saleem, K. & Lodhi, M. A. Secure and privacy-preserving intrusion detection and prevention in the internet of unmanned aerial vehicles. *Sensors* **23** (19), 8077 (2023). 37836907 10.3390/s23198077 PMC10575224

[62] Onsu, A., Kantarci, B. & Boukerche, A. How to Cope with malicious federated learning clients: an unsupervised learning-based approach. *Comput. Netw.* **234**, 109938 (2023).

[63] Popoola, S. I. et al. Federated deep learning for intrusion detection in consumer-centric internet of things. *IEEE Trans. Consum. Electron.* **70**(1), 1610–1622 (2023).

[64] Quyen, Q. H., The, D. P., Chi, V. N., Thu, H. D. T. & Hien, P. V. H. Federated intrusion detection on non-iid data for Iiot networks using generative adversarial networks and reinforcement learning. Cham: Springer International Publishing. *Int. Conf. Inf. Secur. Pract. Experience*, pp. 364–381 (2022).

[65] Bensaid, R., Labraoui, N. & Salameh, H. B. Federated deep learning-based intrusion detection approach for enhancing privacy in fog-iot networks, In 2023 10th Int. Conf. Internet Things: Syst., Manag. Security (IOTSMS), pp. 156–160. (2023).

[66] Rashid, M. et al. A federated learning-based approach for improving intrusion detection in industrial internet of things networks. *Network* **3** (1), 158–179 (2023).

[67] Du, W. et al. TransfficFormer: A Novel Transformer-based Framework To Generate Evasive Malicious Traffic. *Knowl. Based Syst.* **319**, 113546 (2025).

[68] Vellela, S. S. et al. Cyber threat detection in industry 4.0: Leveraging GloVe and self-attention mechanisms in BiLSTM for enhanced intrusion detection. *Comput. Electr. Eng.* **124**, 110368. (2025).

[69] Kumar, M., Singh, S. K. & Kim, S. Hybrid deep learning-based cyberthreat detection and IoMT data authentication model in smart healthcare. *Future Generation Computer Systems*, **166**, 107711. (2025).

[70] Hossain, M. A. & Islam, M. S. *Towards Decentralized Cybersecurity: A Novel Privacy-Preserving Federated Learning Approach for Botnet Attack Detection* p.100355 (Research and Applications, 2025).

[71] Tian, J. et al. *ADMM-based Adversarial False Data Injection Attacks against Multi-Label Locational Detection* (IEEE Transactions on Dependable and Secure Computing, 2025).

[72] Varadarajan, M. N. et al. Smart healthcare data protection and analysis through fuzzy-based cyber security. *J. Environ. Prot. Ecol.* **25** (5), 1604–1614 (2024).

[73] Tkrk, R. & Vekariya & V. Balagoni, Y. Cybersecurity and artificial intelligence for predicting crime rates. *J. Environ. Protect. Ecol*, **25**(5), 1395. (2024).

[74] Rajaram, A. & Palaniswami, D. S. A trust based cross layer security protocol for mobile ad hoc networks. (2009). arXiv:0911.0503.

[75] Rajaram, A. Reputation Based Security Scheme In Dtn.

[76] Mukundan, V., Rajaram, A. & Gopinath, S. Securing mobile ad hoc network using double hash authentication technique. *Int. J. Adv. Inform. Sci. Technol.* **2** (3), 23–28 (2013).

